# Machine Learning-Based QSAR Models for Discovery of Inhibitors Targeting *Leishmania infantum* Amastigotes

**DOI:** 10.3390/ph19040588

**Published:** 2026-04-07

**Authors:** Naivi Flores-Balmaseda, Julio A. Rojas-Vargas, Susana Rojas-Socarrás, Facundo Pérez-Giménez, Francisco Torrens, Juan A. Castillo-Garit

**Affiliations:** 1 Unit of Computer-Aided Molecular ‘‘Biosilico” Discovery and Bioinformatic Research (CAMD-BIR Unit), Departamento de Farmacia, Facultad de Química-Farmacia, Universidad Central ‘‘Marta Abreu” de Las Villas, Santa Clara 54830, Cuba; 2 Doctorado en Informática Aplicada a Salud y Medio Ambiente, Universidad Tecnológica Metropolitana, Ignacio Valdivieso 2409, San Joaquín, Santiago 8940577, Chile; 3 Unidad de Investigación de Diseño de Fármacos y Conectividad Molecular, Departamento de Química Física, Facultad de Farmacia, Universitat de València, 46100 Valencia, Spain; facundo.perez@uv.es; 4 Institut Universitari de Ciència Molecular, Universitat de València, Edifici d’Instituts de Paterna, P.O. Box 22085, E-46071 Valencia, Spain; 5 Instituto Universitario de Investigación y Desarrollo Tecnológico (IDT), Universidad Tecnológica Metropolitana, Ignacio Valdivieso 2409, San Joaquín, Santiago 8940577, Chile

**Keywords:** neglected tropical diseases, machine learning models, *k-means* cluster analysis, *Leishmania infantum*, artificial intelligence, drug discovery

## Abstract

**Background/Objectives**: Leishmaniasis is a group of diseases caused by obligate intracellular parasites of the Leishmania genus and is classified by the World Health Organization as a category I neglected tropical disease. *Leishmania infantum* predominantly affects children under five years of age and shows an increasing incidence of cutaneous and visceral forms. The development of new therapeutic alternatives remains challenging, making in silico approaches valuable for accelerating antileishmanial drug discovery. This study aimed to identify new compounds with potential activity against *Leishmania infantum* amastigotes using artificial intelligence-based classification models. **Methods**: A curated database of compounds with reported biological activity was constructed. Molecular representation employed zero- to two-dimensional descriptors calculated with Dragon software (v 7.0.10). Unsupervised k-means cluster analysis was applied to define training and external prediction sets. Supervised models were developed on the WEKA platform using IBk, J48, multilayer perceptron, and sequential minimal optimization algorithms. Model performance was assessed through internal cross-validation and external validation procedures. **Results**: All models achieved classification accuracies above eighty percent for both training and prediction sets, indicating consistent predictive performance and good generalization ability. The validated models were applied to virtual screening of the DrugBank database and a collection of synthetic compounds. This screening campaign enabled the identification of one hundred twenty compounds with potential activity against the amastigote form of *Leishmania infantum*. **Conclusions**: Artificial intelligence-based QSAR models proved to be useful tools for prioritizing antileishmanial candidates. The integration of molecular descriptors, machine learning, and virtual screening offers an efficient strategy for drug discovery.

## 1. Introduction

Parasites of the genus *Leishmania* are intracellular protozoa belonging to the family *Trypanosomatidae* and are responsible for a group of complex clinical manifestations collectively known as leishmaniasis. This disease is characterized by severe alterations of the host immune system and presents a wide spectrum of clinical outcomes, ranging from self-healing cutaneous lesions to fatal visceral infections [1,2]. According to the World Health Organization, leishmaniasis is classified as a neglected tropical disease (NTD), predominantly affecting tropical and subtropical regions and impacting more than one billion people annually [3]. Its designation as “neglected” arises from the limited attention it has historically received, as it mainly affects low-income populations in developing countries with restricted access to adequate healthcare systems [4]. In recent years, leishmaniasis has gained increased relevance due to a marked rise in its global incidence and geographical expansion, currently being endemic in 98 countries across five continents, with approximately 350 million people living in areas at risk of infection [3,5].

The genus *Leishmania* comprises 53 recognized species, of which 29 are distributed in the Old World (southern Europe, Africa, the Middle East, Central Asia, and the Indian subcontinent), 20 in the New World (Central and South America), three are present in both regions, and only one species has been reported in Australia [6]. Among these, 31 species are pathogenic to mammals, and 20 are directly responsible for the diverse clinical forms of leishmaniasis in humans [6,7]. The parasite exhibits two main morphological forms during its life cycle: the extracellular, flagellated promastigote, predominant in the insect vector, and the intracellular, non-flagellated amastigote, which proliferates within vertebrate host cells [8,9]. Transmission occurs through the bite of infected female hematophagous dipterans of the genus *Phlebotomus* in the Old World and *Lutzomyia* in the New World [10,11]. Due to its zoonotic nature, leishmaniasis affects both humans and domestic animals, particularly dogs, which constitute the principal urban reservoir, alongside several wild mammalian species [10]. The complex biological cycle of *Leishmania*, involving multiple parasite species, vectors, and reservoirs, gives rise to a broad range of clinical syndromes affecting the skin, mucosae, and internal organs [12]. In the Americas, the disease presents with high prevalence and wide distribution, further exacerbated by social, economic, and environmental risk factors that significantly increase population vulnerability [13].

Among the pathogenic species, *Leishmania infantum* is of particular importance due to its presence in both the Old and New Worlds and its role as the etiological agent of zoonotic visceral leishmaniasis [12,14]. This species primarily infects macrophages in hematopoietic organs such as the bone marrow, spleen, and liver, leading to severe organ dysfunction [11]. In humans, infection by *L. infantum* may result in clinical presentations ranging from cutaneous leishmaniasis to the highly lethal visceral form. Although leishmaniasis was traditionally considered a rural disease, it is now well established in urban environments, where domestic dogs act as the main reservoir [13]. The discovery of new antileishmanial compounds remains a major scientific challenge. The diversity of *Leishmania* species, the parasite’s complex life cycle, and the heterogeneity of clinical manifestations hinder the identification of effective lead compounds [15]. Moreover, current therapeutic options are limited by high toxicity, severe adverse reactions, prolonged treatment regimens, and the emergence of drug resistance [16,17]. These limitations are further aggravated by an increasing parasite burden, the risk of HIV co-infection, and reduced drug responsiveness due to suboptimal dosing strategies [18].

Although no autochthonous cases of leishmaniasis have been reported in Cuba, the disease is of considerable national interest due to its global epidemiological relevance, the country’s geographical position, and the internationalist principles of its public health system [19]. Additionally, recent scientific and technological advances have introduced novel approaches for addressing unresolved aspects of leishmaniasis management [20]. In this context, there is an urgent need to discover new molecular entities with antileishmanial activity that exhibit improved therapeutic indices, reduced toxicity, and lower susceptibility to resistance [16]. To date, no compounds fully meeting these criteria have been identified, and traditional drug discovery approaches based on trial-and-error remain time-consuming and resource-intensive [15].

Computer-aided drug design (CADD) offers a faster and more cost-effective alternative for the discovery and optimization of bioactive compounds [21]. In silico methodologies encompass a wide range of computational techniques used to support drug discovery, with quantitative structure–activity/property relationship (QSAR/QSPR) modeling playing a central role [22,23]. These approaches have significantly contributed to the development of several drugs currently available on the market [24]. At the Faculty of Chemistry and Pharmacy of the Universidad Central “Marta Abreu” de Las Villas, the Computer-Aided Molecular Design and Bioinformatics Research Group (CAMD-BIR Unit) has reported relevant advances in the application of computational and graph-theoretical QSAR methodologies for the rational design of potentially bioactive organic compounds [25,26,27,28,29].

In a previous study, we developed predictive models for antileishmanial activity against *Leishmania amazonensis* using curated datasets and computational approaches [30]. However, the present study focuses on *Leishmania infantum*, a parasite species with important biological and clinical differences. While *L. amazonensis* is mainly associated with cutaneous and diffuse cutaneous leishmaniasis, *L. infantum* is one of the principal causative agents of visceral leishmaniasis, a systemic and potentially fatal disease that affects internal organs such as the spleen, liver, and bone marrow [5,31,32]. These differences imply distinct pharmacological contexts, including differences in therapeutic strategies, drug distribution requirements, and efficacy endpoints. Importantly, several studies have shown that susceptibility to antileishmanial drugs varies significantly among *Leishmania* species, with substantial interspecies differences in response to pentavalent antimonials, amphotericin B, and other compounds [15,16]. Such variability is associated with species-specific biological traits, and molecular mechanisms of drug response and resistance, including differential gene expression patterns linked to antimonial resistance in *L. amazonensis* [33]. Consequently, structure–activity relationships derived from datasets generated against one *Leishmania* species cannot be assumed to be directly transferable to another. In addition, the chemical space explored in the present dataset differs from that used in the previous study, as it includes compounds experimentally evaluated against *L. infantum* amastigotes, which may lead to different distributions of biological activity and relevant molecular descriptors. Consequently, the resulting structure–activity relationships may differ between datasets derived from different parasites [15,16]. Therefore, it is necessary to develop species-specific predictive models in order to capture the pharmacological and molecular determinants associated with activity against *L. infantum*, and to identify compounds with potential efficacy against visceral leishmaniasis, which remains one of the most severe and potentially fatal forms of the disease.

Based on the aforementioned considerations, the scientific problem addressed in this study is the limited efficacy and safety of current drugs used to treat leishmaniasis caused by *Leishmania infantum*, which necessitates the discovery of new active molecular entities. We hypothesize that it is possible to identify novel compounds with potential antileishmanial activity against *L. infantum* through the application of QSAR methodologies combined with artificial intelligence techniques [22,34]. Accordingly, the main objective of this work is to identify new antileishmanial compounds using in silico approaches.

## 2. Results and Discussion

### 2.1. Data Management, Curation, and Chemical Space Coverage

The construction of a robust and predictive QSAR model critically depends on the quality, consistency, and representativeness of the underlying dataset. In this study, an initial collection of 934 compounds was retrieved from PubChem bioassays reporting activity against Leishmania species. Following a rigorous curation process aimed at minimizing experimental noise and annotation inconsistencies, a final dataset of 437 compounds active against *Leishmania infantum* amastigotes was obtained, comprising 202 active and 235 inactive molecules. Activity classification was primarily based on IC_50_ values while also considering reported mechanisms of action and structural patterns, thereby reducing the risk of mislabeling compounds tested under heterogeneous experimental conditions.

**Figure 1:**
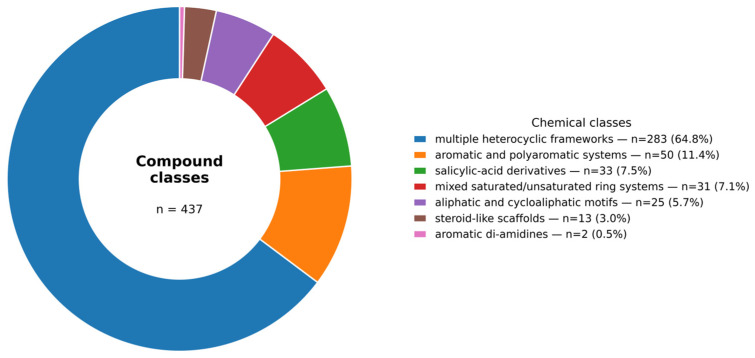
Distribution of compounds across major chemical classes in the dataset.

**Figure 2:**
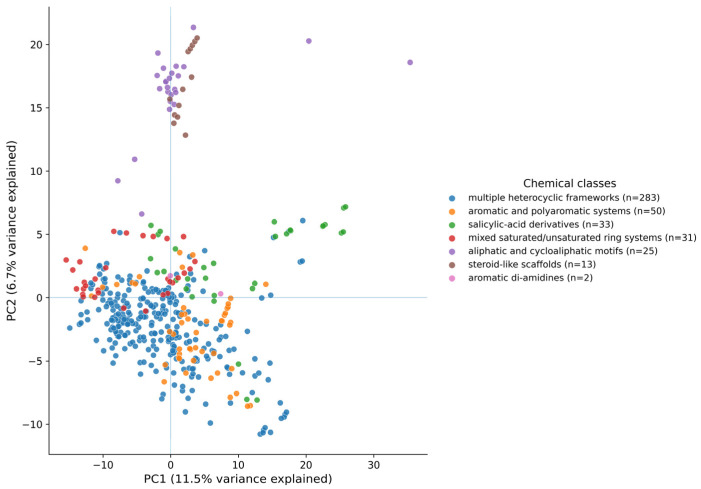
Chemical space coverage of the dataset visualized by principal component analysis (PCA).

The curated dataset exhibits a high degree of chemical and structural diversity, including multiple heterocyclic frameworks, aromatic and polyaromatic systems, aliphatic and cycloaliphatic motifs, steroid-like scaffolds, salicylic-acid derivatives, aromatic diamidines, substituted pyrimidines, and mixed saturated/unsaturated ring systems. The distribution of compounds in these major chemical classes is illustrated in Figure 1. As shown in the figure, the dataset is not dominated by a single type of scaffold but instead includes multiple structural classes, which supports the chemical heterogeneity of the compound collection. Such diversity is essential for developing QSAR models with broad applicability domains, particularly in neglected disease research, where chemical space exploration remains limited. The structural heterogeneity observed here provides a solid foundation for machine learning-based classification and reduces the likelihood of model bias toward narrow scaffold families. This structural diversity is further supported by the PCA-based chemical space analysis (Figure 2), which shows that the compounds occupy a large region of the descriptor space used for the development of the model.

Molecular representation was achieved through the calculation of 2489 zero- to two-dimensional molecular descriptors using DRAGON software [35]. These descriptors encompass constitutional properties, substructure fragment counts, functional group frequencies, topological indices, and connectivity-related parameters, collectively capturing both global and local molecular features. To mitigate multicollinearity and reduce the risk of overfitting, a correlation filter (|r| > 0.9) was applied, resulting in a reduced set of 1187 non-redundant descriptors. This dimensionality reduction step is particularly important for classification problems involving moderately sized datasets, as excessive descriptor redundancy can artificially inflate model performance while degrading generalizability.

### 2.2. Diversity-Preserving Dataset Splitting Strategy

To ensure that the predictive performance of the models reflects genuine generalization rather than favorable data partitioning, a diversity-driven splitting strategy was implemented. Instead of using random or purely stratified splits, cluster analysis was performed separately for active and inactive compounds using k-means cluster analysis (k-MCA) implemented in STATISTICA 8.0 [36], following previously established recommendations [37,38]. This approach ensures that each subset contains representative chemical diversity from the full dataset, thereby minimizing the risk of “easy splits” where structurally similar compounds are disproportionately grouped.

**Figure 3:**
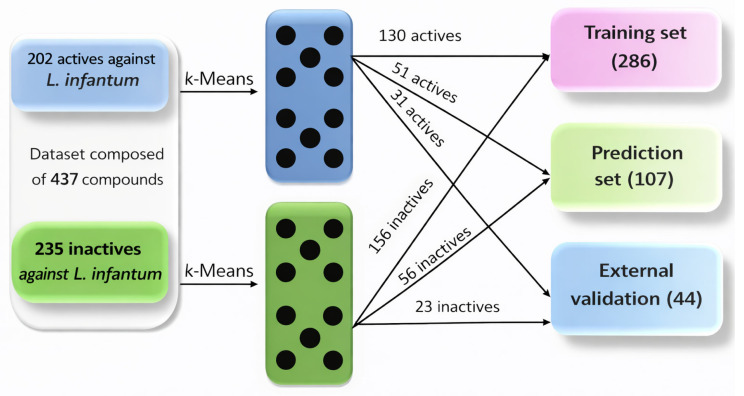
Workflow describing the algorithm used to design the training, prediction, and external validation sets based on *k-means cluster analysis* (k-MCA).

As illustrated in Figure 3, the clustering-based strategy resulted in 286 compounds assigned to the training set, 107 compounds to the prediction set, and 44 compounds to an external validation set. This design strengthens the methodological rigor of the study, as it forces the models to learn structure–activity relationships that are transferable across different regions of chemical space. From a QSAR validation standpoint, this step represents a key strength of the work and directly addresses common reviewer concerns regarding dataset bias and overoptimistic performance estimates.

### 2.3. Descriptor Selection and Algorithm-Specific Model Optimization

Given the heterogeneity of the descriptor space and the fundamentally different learning principles underlying each classification algorithm, descriptor selection was carried out independently for each model using the WEKA platform. Multiple evaluators, filters, and search strategies were applied to identify compact yet informative subsets of descriptors tailored to each algorithm.

Starting from the initial pool of 1187 filtered descriptors, preliminary reduced subsets were obtained for each classifier. These initial subsets consisted of 18 descriptors for IBk, 13 for J48, 20 for MLP, and 33 for SVM. These subsets were subsequently refined to obtain the final descriptor sets used for model construction. This algorithm-specific feature selection strategy is particularly important in comparative QSAR studies, as enforcing a single descriptor subset across heterogeneous machine learning algorithms often leads to suboptimal model performance and potentially misleading comparisons.

The novelty of the present modeling work is further emphasized by the limited availability of comparable classification studies based on artificial intelligence targeting *Leishmania* species. In this context, the work of Flores-Balmaseda et al. (2015) [39] represents the primary benchmark for comparison. The present study extends upon previous efforts by incorporating a carefully curated dataset, multiple layers of validation, and consensus modeling strategies.

After selecting the descriptors, the hyperparameters of each machine learning algorithm were optimized using the WEKA platform. For each classifier, a batch exploration of multiple combinations of parameters within predefined ranges was performed. The model optimization was performed using only the training set. The external test set was not used in the model optimization process and was reserved exclusively for the final evaluation of predictive performance. The use of an independent external validation set follows the best practices recommended for the development of QSAR models and provides a robust assessment of the predictive performance of the models, in accordance with the validation principles proposed by the Organization for Economic Co-operation and Development (OECD) [40].

The final models were built using optimized hyperparameters specific to each algorithm. The IBk classifier was configured with k = 3 nearest neighbors using the Manhattan distance. The J48 decision tree was generated using a pruning confidence factor of 0.09 and a minimum of five instances per leaf. The MLP model consisted of a single hidden layer containing 13 neurons and was trained for 500 epochs with a learning rate of 1.0 and momentum of 0.8. The SVM model was implemented using the SMO (sequential minimal optimization) algorithm with a radial basis function (RBF) kernel, with cost (C) and gamma parameters optimized during model development. The structural characteristics and hyperparameters of the developed models are summarized in Table S2 (Supporting Information), while the complete WEKA command-line configurations used for model training and testing are detailed in Table S1.

### 2.4. Classification Model Performance on Training and Prediction Sets

#### 2.4.1. IBk (k-Nearest Neighbors) Model

The IBk classifier demonstrated the strongest overall performance among the evaluated models. As reported in Table 1, the model achieved 88.81% accuracy on the training set and 85.05% accuracy on the prediction set, with ROC-AUC values of 0.955 and 0.863, respectively. Importantly, sensitivity and specificity remained well balanced across both sets, indicating that the model does not favor one class disproportionately.

The low false-positive rates observed further support the suitability of the IBk model for antileishmanial activity classification. The descriptors used in this model reveal that topological descriptors, atom-centered fragments, and 2D autocorrelation indices play a central role in defining the local similarity relationships exploited by the kNN algorithm. Compared to the reference model by Flores-Balmaseda et al. [39], the present IBk model exhibits improved training performance while maintaining competitive predictive accuracy, highlighting the benefits of dataset curation and descriptor optimization.

#### 2.4.2. J48 Decision Tree Model

The J48 decision tree model achieved 87.76% training accuracy and 84.11% prediction accuracy, with ROC-AUC values exceeding 0.88 (Table 2). A particularly notable result is the high training specificity (91.30%), indicating a strong capacity to correctly identify inactive compounds. This property is advantageous in early-stage virtual screening, where reducing false positives can significantly lower experimental costs.

Descriptor analysis indicates that constitutional descriptors, connectivity indices, and functional group counts dominate the decision process, enabling the extraction of interpretable structure–activity rules. From a scientific perspective, this interpretability adds value beyond raw predictive performance, as it facilitates hypothesis generation and guides rational compound optimization.

#### 2.4.3. MLP Neural Network Model

The MLP model displayed stable and consistent performance across training and prediction sets, with accuracies of 83.22% and 83.18%, respectively (Table 3). Although slightly less accurate than IBk and J48, the MLP achieved competitive ROC-AUC values and demonstrated a more conservative classification profile, characterized by higher specificity than sensitivity in the prediction set.

This conservative classification behavior is advantageous when the objective is to prioritize compounds with a higher confidence of true biological activity, particularly in early-stage screening, where minimizing false-positive selections is critical. The descriptor analysis suggests that the neural network captures complex nonlinear relationships among topological and information-based descriptors. Notably, the MLP model outperforms the corresponding benchmark reported by Flores-Balmaseda et al. [39], supporting the consistency of the present modeling framework.

#### 2.4.4. SVM (Support Vector Machine) Model

The SVM model yielded the lowest overall accuracy among the evaluated classifiers (81.47% training; 78.50% prediction, Table 4) but clearly excelled in terms of specificity and false-positive minimization. Training and prediction specificities exceeded 91%, with false-positive rates below 6%.

**Figure 4:**
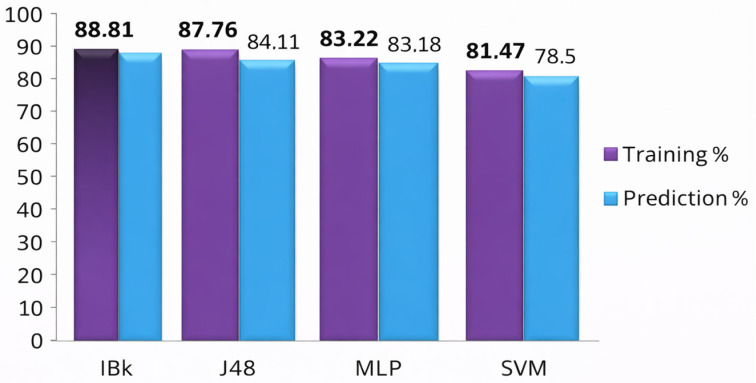
Comparison of correct classification percentages for training and prediction sets across all models.

These results highlight the precision-oriented nature of the SVM classifier, which is particularly valuable in contexts where experimental validation resources are limited. Descriptors used in this model indicate a strong reliance on connectivity indices and functional group frequency descriptors, consistent with the margin-based decision boundaries characteristic of SVMs. As summarized in Figure 4, the SVM model complements the higher-accuracy classifiers by providing a highly conservative screening filter.

### 2.5. External and Internal Validation of Model Robustness

**Figure 5:**
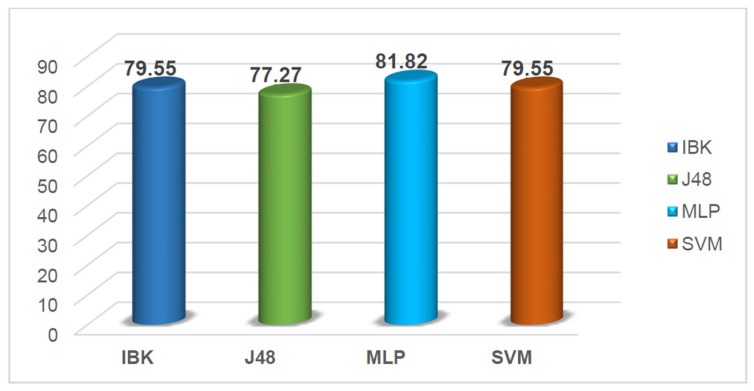
Comparison of correct classification percentages in the external validation set.

The primary criterion for the acceptance or rejection of a classification model relies on its performance on the external prediction set, which reflects the model’s predictive performance on unseen compounds. As previously described, the performance of each developed machine learning (ML) model was systematically evaluated using both validation and external datasets. The comparative results obtained for all models are summarized in Table 5. External validation using an independent set of 44 compounds provided an additional assessment of the predictive behavior of the developed models. As reported in Table 5 and Figure 5, the MLP model achieved the highest external accuracy (81.82%), indicating a relatively balanced classification performance. In contrast, the SVM model achieved 100% specificity, correctly classifying all inactive compounds in the external set. However, this result should be interpreted with caution due to the relatively small size of the external dataset. Moreover, the high specificity was accompanied by a lower sensitivity (57.14%) and a moderate MCC (0.64), indicating that the model tends to favor inactive predictions and may generate a higher number of false negatives. Therefore, rather than indicating apparent high predictive performance, the observed performance suggests that the SVM model prioritizes minimizing false positives. This behavior may be advantageous in screening scenarios where avoiding false positives is critical, but it also highlights the importance of considering complementary models with more balanced sensitivity–specificity profiles. According to the OECD principles for the validation of QSAR models, model performance should be interpreted by considering multiple statistical metrics rather than a single indicator [40].

Internal validation was conducted using *10-fold cross-validation* on the training set, following established best practices [41,42]. The results summarized in Table 6 and Table 7 demonstrate that all models maintain stable performance across folds, with no evidence of severe overfitting. Notably, the SVM model exhibits the smallest discrepancies between training and cross-validation metrics, leading the authors to identify it as the most robust and reproducible model overall, despite its lower raw accuracy.

The comparative performance of the four machine learning classifiers highlights distinct trade-offs between predictive accuracy, sensitivity, and specificity (Table 7). The IBk model exhibited the highest overall quality (Q) under cross-validation (88.81%), accompanied by a strong balance between sensitivity (86.92%) and specificity (88.28%), indicating a robust ability to correctly classify both active and inactive compounds. However, its external sensitivity decreased notably (76.92%), together with an increased false-positive rate (FPR = 23.08%), suggesting a reduced generalization capability when applied to unseen data. In contrast, the J48 decision tree demonstrated a more conservative behavior, reflected in a lower cross-validation Q value (78.67%) but a markedly improved specificity in the external set (91.30%) and a reduced FPR (6.41%), which is advantageous for minimizing false-positive predictions. The MLP model showed moderate and more homogeneous performance across validation schemes, although it presented the highest FPR during cross-validation (23.08%), indicating a tendency to overpredict active compounds. Finally, the SVM classifier achieved a favorable compromise between specificity and error control, yielding the lowest FPR in both cross-validation (7.05%) and external validation (3.85%), albeit at the expense of lower sensitivity, particularly in the external set (63.85%). Overall, these results emphasize that while IBk achieves the highest classification performance, SVM and J48 provide more stringent classification with reduced false positives, which may be preferable in virtual screening campaigns where reliability and experimental cost reduction are critical.

### 2.6. Virtual Screening and Consensus-Based Hit Prioritization

**Figure 6:**
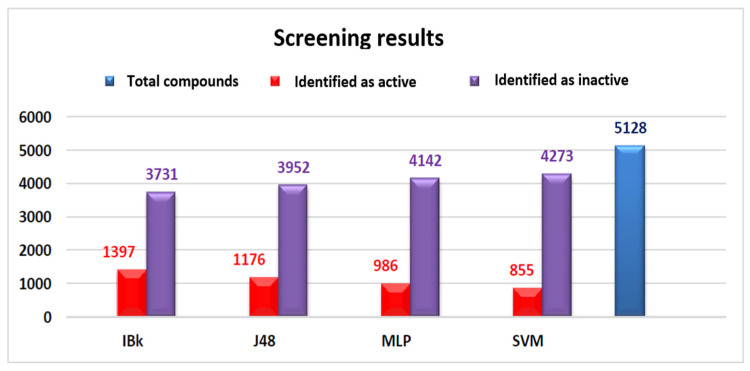
Number of predicted active and inactive compounds for each classification model in the virtual screening.

The validated models were subsequently applied to a virtual screening campaign involving 5128 compounds, including 4660 DrugBank molecules [43,44,45] and 468 synthetic compounds from collaborating laboratories. Individual model predictions varied substantially, as shown in Figure 6, reflecting the distinct decision strategies of each classifier.

**Figure 7:**
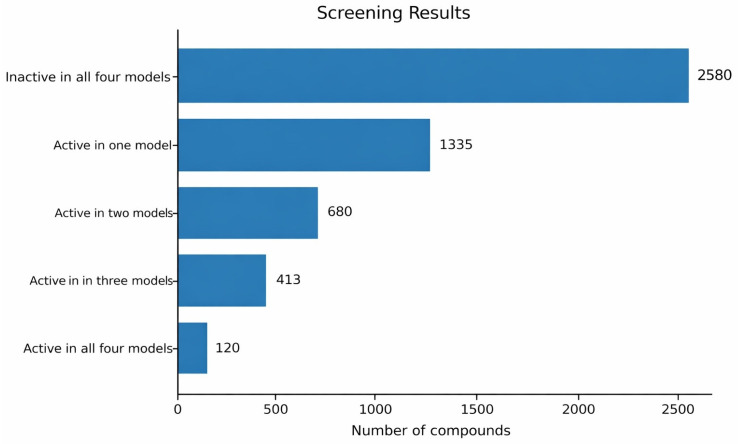
Distribution of compounds according to the number of machine learning models that predict them as active during the virtual screening process.

To increase confidence in the predicted hits, a consensus modeling approach was adopted. As summarized in Figure 7, 1335 compounds were predicted active by at least one model, while 120 compounds were predicted active by all four models, including 115 DrugBank compounds and five synthetic candidates. This progressive reduction highlights the effectiveness of consensus modeling in prioritizing a manageable and high-confidence subset of candidates for experimental validation, substantially reducing the number of compounds that would otherwise require experimental screening.

From a translational standpoint, the identification of DrugBank compounds within the four-model consensus is particularly significant, as these molecules may benefit from existing pharmacokinetic and safety data, thereby accelerating downstream experimental and clinical evaluation.

An examination of the descriptors selected across the different machine learning models provides insight into the structural features associated with antileishmanial activity. Several models include Kier–Hall valence connectivity indices (e.g., X0Av, X1Av, X2v, and X3Av), which describe molecular size, branching patterns, and overall topological complexity. These descriptors are usually related to physicochemical properties that influence membrane permeability and the ability of compounds to interact with biological targets in protozoan parasites.

Information theory-based descriptors, such as SIC5 and CIC5, capture aspects of molecular symmetry and structural diversity, suggesting that the spatial distribution of atoms and substituents may influence biological activity. In addition, Burden matrix eigenvalues (e.g., SpMin4_Bh(m), SpMax1_Bh(m), SpMin1_Bh(v), and SpMin4_Bh(e)) encode electronic and steric properties derived from atomic masses, electronegativity, polarizability, and van der Waals volumes, which are often associated with electronic distribution and intermolecular interactions involved in ligand–target binding.

Fragment-based descriptors and functional group counts further highlight chemically meaningful features within the dataset. In particular, descriptors associated with tertiary amines, amidine-type functionalities, and halogenated fragments indicate that specific chemical groups may contribute to activity by modulating lipophilicity, polarity, hydrogen-bonding capacity, and electrostatic interactions with biological targets.

Interestingly, several of the functional groups captured by the selected descriptors are also present in compounds identified among the predicted hits during the virtual screening, including molecules containing amine-based functionalities, heteroaromatic systems, and highly polar phosphate-containing groups such as those present in bisphosphonates.

It is important to note that the predicted hits include compounds belonging to several pharmacologically relevant categories, such as approved drugs, bioactive molecules in the investigational phase, synthetic compounds, and endogenous metabolites. The presence of approved drugs among the predicted compounds highlights the pharmacological diversity of the screened dataset and supports the biological plausibility of the computational predictions.

Among the approved drugs identified within the consensus set are disulfiram (DB00822), an aldehyde dehydrogenase inhibitor used for the treatment of alcohol use disorder, and several nitrogen-containing bisphosphonates such as alendronate (DB00630) and pamidronic acid (DB00282), which are widely used in the treatment of osteoporosis and disorders associated with increased bone resorption. The identification of bisphosphonate derivatives is particularly noteworthy because this class of compounds has previously demonstrated antiparasitic activity. In particular, aromatic bisphosphonate derivatives have been reported to inhibit parasite replication in *Trypanosoma*, *Leishmania*, *Toxoplasma*, and *Plasmodium*, in some cases exhibiting IC_50_ values in the nanomolar to low micromolar range [46].

Among the predicted compounds, other approved or clinically used drugs have been identified, such as the antiviral agent foscarnet (DB00529), the antineoplastic drugs pipobroman (DB00236) and busulfan (DB01008), the farnesyltransferase inhibitor lonafarnib (DB06448), and the marine-derived anticancer compound trabectedin (DB05109). The presence of multiple drugs with well-characterized pharmacological profiles highlights the potential opportunities for drug repurposing in the discovery of antileishmanial drugs.

The dataset also includes several bioactive molecules in the research or experimental phase that are currently under clinical or preclinical evaluation. Examples include 1-oleoyl-2-palmitoylphosphatidylcholine (DB05456), which has been investigated as a therapeutic candidate for acute coronary syndromes, QS-21 (DB05400), an immunological adjuvant evaluated in several clinical trials, and bevirimat (DB06581), which has been explored as a maturation inhibitor for HIV treatment. The presence of these molecules further illustrates the chemical and pharmacological diversity detected through computational screening.

In addition to the DrugBank-derived molecules, five synthetic compounds from collaborating laboratories were also predicted as active by all four models. One of these molecules contains a halogenated heteroaromatic core bearing two *tert*-butyl-substituted phenyl groups, a scaffold that combines high lipophilicity with electron-withdrawing substituents that may improve membrane permeability and protein binding. The remaining compounds share a triarylmethane-based scaffold functionalized with dimethylamino-substituted aromatic rings and a glycosyl moiety.

Triarylmethane derivatives are well known for their antimicrobial and antiparasitic properties, while the presence of dimethylamino aromatic groups may facilitate interactions with biological targets [47,48]. In addition, glycosylation and acetylation patterns may modulate physicochemical properties such as solubility, lipophilicity, and cellular uptake [49]. The identification of these compounds by all four models highlights their potential as promising candidates for further experimental evaluation.

Furthermore, the set of predicted hits contains small molecules with diverse structures and heterocyclic scaffolds commonly found in antimicrobial and antiparasitic drug discovery libraries. This structural diversity suggests that the models can identify compounds spanning multiple regions of biologically relevant chemical space, an important requirement for the discovery of structurally diverse antileishmanial candidates. Taken together, these descriptors suggest that antileishmanial activity in the analyzed dataset is influenced by a combination of molecular topology, electronic properties, and pharmacologically relevant functional groups.

From a translational perspective, it is important to recognize that drug discovery is inherently a multi-stage process in which computational predictions, in vitro assays, and in vivo studies provide complementary information. While experimental validation is essential, early-stage computational prioritization plays a critical role in reducing the number of candidate compounds and guiding subsequent biological evaluation. In this context, the present framework enables the identification of a reduced set of high-confidence candidates from large chemical libraries, thereby supporting more efficient downstream experimental efforts.

Therefore, the compounds prioritized in this study represent testable hypotheses that can guide future experimental validation efforts aimed at identifying novel antileishmanial agents.

Taken together, these results demonstrate that the integration of diversity-aware data splitting, algorithm-specific descriptor selection, multi-level validation, and consensus modeling yields a consistent and scientifically sound QSAR framework capable of supporting the identification and prioritization of novel antileishmanial candidates. The complementary strengths of the evaluated classifiers—accuracy-driven methods (IBk and J48), a balanced neural network model (MLP), and a margin-based support vector machine (SMO)—justify their combined use in virtual screening pipelines and provide a rational basis for prioritizing compounds for experimental testing.

## 3. Materials and Methods

### 3.1. Data Collection and Curation

All compounds included in this study, together with their biological activity profiles, were compiled from publicly available PubChem BioAssay records reporting experimental evaluations against *Leishmania infantum* amastigotes. These assays correspond to studies published in peer-reviewed, high-impact journals indexed in the Web of Science database, primarily between 1983 and 2016. When a compound appeared in more than one source, IC_50_ values derived from the most rigorous and clearly described experimental methodologies were selected. Additional metadata were extracted, including parasite life stage, assay conditions, reported IC_50_ values, molecular weight, and chemical structures derived from canonical SMILES representations. Compounds were classified as active when IC_50_ ≤ 1.5 μM and inactive otherwise, following thresholds commonly applied in antileishmanial screening studies [43,45,50].

### 3.2. Chemical Structure Representation and Curation

A total of 437 chemical structures were included in the dataset. Most structures were retrieved directly from PubChem [45,50], while the remaining compounds were manually drawn using ChemDraw Ultra 8.0 [51] and exported as *.sdf as MDL files. All structures were subsequently curated and standardized using Open Babel 2.4.1 [52], including the addition of explicit hydrogen atoms, charge neutralization, and removal of salts or counterions that could affect descriptor calculation. Duplicate structures were identified and removed using ISIDA [53] and EdiSDF [54], ensuring a non-redundant chemical dataset suitable for quantitative modeling.

### 3.3. Molecular Descriptor Calculation

Molecular descriptors encoding structural, physicochemical, and topological information were calculated using DRAGON Professional software v.7.0.10 [35]. Descriptor families spanning 0D to 3D representations were generated, resulting in an initial pool of 1 187 descriptors. Constant, near-constant descriptors and highly correlated variables (Pearson correlation coefficient > 0.90) were eliminated to reduce redundancy and minimize the risk of overfitting. Descriptor matrices were organized in spreadsheet format, with compounds represented as rows and descriptors as columns, supported by JChem for Excel for structure handling and visualization [23].

### 3.4. Statistical Analysis and Data Partitioning

To ensure adequate structural diversity and representative sampling, cluster analysis was performed separately for active and inactive compounds using k-means cluster analysis (k-MCA) implemented in STATISTICA 8.0 software [36]. Prior to clustering, all descriptor matrices were standardized. Compounds were randomly selected from each cluster to construct three non-overlapping subsets: a training set (65%), a prediction/test set (25%), and an external validation set (10%). This strategy ensured that chemical classes defined by clustering were proportionally represented across all subsets. Compounds included in the prediction and external validation sets were not used during model training [55,56].

### 3.5. Feature Selection and Machine Learning Modeling

QSAR classification models were developed using the WEKA 3.6 data mining platform [57]. As previously stated, the initial pool of calculated molecular descriptors was used as the set of input attributes after standard preprocessing steps, which included removing constant or near-constant descriptors and highly correlated variables. Feature selection was performed using the feature selection tools available in WEKA, combining filter-based and wrapper-based approaches. Multiple attribute evaluators and search strategies were explored for each learning algorithm to identify compact and informative subsets of descriptors that would maximize model performance. Four supervised learning techniques were evaluated: k-nearest neighbors (IBk) [58], decision trees (J48) [59], multilayer perceptron neural networks (MLPs) [60] and support vector machines (SVMs) [61]. It is important to note that the descriptor selection and model optimization procedures were performed using only the training set. The external prediction set was not used during descriptor selection or model development and was reserved exclusively for the final evaluation of the model’s predictive performance. This workflow follows the best practices recommended for the development and validation of QSAR models [30,62].

### 3.6. Model Validation and Performance Evaluation

Final models were selected according to the principle of parsimony, favoring those with the highest statistical significance and predictive performance using the smallest possible number of descriptors. A complete list of the molecular descriptors selected for each machine learning model is provided in the Supplementary Material (Table S3). Model performance was assessed using standard classification metrics, including overall accuracy (Q), sensitivity, specificity, false-positive rate (FPR), and the Matthews correlation coefficient (MCC). Internal validation was performed using 10-fold cross-validation, while predictive power was further assessed using both the independent prediction set and an external validation set composed of compounds not involved in model construction. These validation strategies are consistent with widely accepted recommendations for robust QSAR modeling [22,40,63].

### 3.7. Virtual Screening and Hit Identification

The DrugBank database was employed as the primary source for virtual screening. DrugBank integrates bioinformatic and chemoinformatic data for FDA-approved drugs, experimental compounds, and associated protein targets [64]. The database was screened using the validated QSAR models to identify compounds with potential activity against *L. infantum* amastigotes.

Additionally, a set of 468 synthetic compounds obtained from organic chemistry laboratories, 407 from the University of Rostock (Germany), and 61 from the Conservatoire National des Arts et Métiers (CNAM, France) was screened. The best identified hits were structurally analyzed and reported as promising candidates for future antileishmanial drug discovery efforts.

## 4. Conclusions

In this study, we developed and validated a set of machine learning-based QSAR models for the identification of compounds with potential activity against *Leishmania infantum* amastigotes. By integrating rigorous data curation, diversity-aware dataset splitting, algorithm-specific descriptor selection, multi-level validation, and consensus-based virtual screening, we established a robust and interpretable computational framework for the prioritization of antileishmanial candidates.

The application of this framework to a dataset of more than 5000 compounds enabled the identification of a reduced set of high-confidence candidates, including DrugBank molecules with known pharmacological profiles and structurally diverse synthetic compounds. The pharmacological interpretation of the predicted hits and the analysis of relevant molecular descriptors provide additional support for the biological plausibility of the results.

Importantly, the proposed approach is not intended to replace experimental validation, but to support early-stage drug discovery by focusing experimental efforts on the most promising candidates. In this context, the compounds identified in this study represent experimentally testable hypotheses that may contribute to the discovery of novel antileishmanial agents. Future work will focus on the experimental evaluation of these prioritized compounds, including in vitro assays against Leishmania infantum amastigotes, to further assess their biological relevance.

Overall, this work highlights the value of descriptor-based machine learning and consensus modeling as practical tools for antiparasitic drug discovery, particularly in scenarios where efficient prioritization strategies are essential to reduce experimental costs and accelerate the identification of candidate compounds.

## Figures and Tables

**Table 1:** Statistical parameters of the IBk model for training and prediction sets.

Set	C	Q (%)	Sensitivity (%)	Specificity (%)	FPR (%)
**Training**	0.77	88.81	86.92	85.71	3.01
**Prediction**	0.70	85.05	80.39	73.33	7.84

**C:** Mathews correlation coefficient, **Q:** accuracy, **FPR:** false-positive rate.

**Table 2:** Statistical parameters for the J48 model (classification tree).

Set	C	Q (%)	Sensitivity (%)	Specificity (%)	FPR (%)
**Training**	0.76	87.76	86.92	85.71	3.01
**Prediction**	0.68	84.11	80.39	73.33	7.84

**C:** Mathews correlation coefficient, **Q:** accuracy, **FPR:** false-positive rate.

**Table 3:** Statistical parameters obtained for the MLP (multilayer perceptron) model.

Set	C	Q (%)	Sensitivity (%)	Specificity (%)	FPR (%)
**Training**	0.76	87.76	86.92	85.71	3.01
**Prediction**	0.68	84.11	80.39	73.33	7.84

**C:** Mathews correlation coefficient, **Q:** accuracy, **FPR:** false-positive rate.

**Table 4:** Statistical parameters obtained for the SVM (support vector machine) model.

Set	C	Q (%)	Sensitivity (%)	Specificity (%)	FPR (%)
**Training**	0.65	81.47	63.85	93.26	3.85
**Prediction**	0.59	78.50	60.78	91.18	5.36

**C:** Mathews correlation coefficient, **Q:** accuracy, **FPR:** false-positive rate.

**Table 5:** External validation statistical parameters for the four classification models.

Set	C	Q (%)	Sensitivity (%)	Specificity (%)	FPR (%)
**IBk**	0.59	79.55	71.43	83.33	13.04
**J48**	0.54	77.27	71.43	78.95	17.39
**MLP**	0.64	81.82	71.43	88.24	8.7
**SVM**	0.64	79.55	57.14	100	0

**C:** Mathews correlation coefficient, **Q:** accuracy, **FPR:** false-positive rate.

**Table 6:** Internal validation statistical parameters for the four classification models.

Set	C	Q (%)	Sensitivity (%)	Specificity (%)	FPR (%)
**IBk**	0.54	76.92	76.92	73.53	23.08
**J48**	0.57	78.67	70.77	80.00	14.74
**MLP**	0.50	75.35	73.44	72.31	23.08
**SVM**	0.58	78.32	60.77	87.78	7.05

**C:** Mathews correlation coefficient, **Q:** accuracy, **FPR:** false-positive rate.

**Table 7:** Internal validation statistical parameters for the four classification models.

Models		Accuracy (%)	Sensitivity (%)	Specificity (%)	FPR (%)
**IBK**	Training set	88.81	86.92	88.28	9.62
	*10-fold-cv*	76.92	76.92	73.53	23.08
**J48**	Training set	87.76	80.77	91.30	6.41
	*10-fold-cv*	78.67	70.77	80.00	14.74
**MLP**	Training set	83.22	79.23	83.06	13.46
	*10-fold-cv*	75.35	73.44	72.31	23.08
**SVM**	Training set	81.47	63.85	93.26	3.85
	*10-fold-cv*	78.32	60.77	87.78	7.05

## Data Availability

The original contributions presented in this study are included in the article/Supplementary Material.

## Supplementary Materials

The following supporting information can be downloaded at https://www.mdpi.com/article/10.3390/ph19040588/s1, Figure S1: Representative consensus hits predicted as active by all four machine-learning models during the virtual screening; Table S1: Final machine learning model configurations implemented in WEKA 3.6; Table S2: Structural characteristics and main hyper parameters of the machine learning models used for QSAR classification; Table S3: Molecular descriptors used in each machine-learning model developed in this study; Excel File S1: curated database and predictions for each serie.
