# Supplementary material for: Machine Learning-Based QSAR Models for Discovery of Inhibitors Targeting Leishmania infantum Amastigotes
Source: Pharmaceuticals (Basel). 2026 Apr 7;19(4):588. doi: 10.3390/ph19040588 (PMC13118347; doi:10.3390/ph19040588)
Supplement: Supplementary file 1 [file pharmaceuticals-19-00588-s001.zip › Supplementary Material.pdf]

Supplementary Material

Machine Learning–Based QSAR models for discovery of inhibitors targeting *Leishmania infantum* amastigotes

Naivi Flores-Balmaseda[1,†], Julio A. Rojas-Vargas[2,†], Susana Rojas-Socarrás[1], Facundo Perez-Gimenez,[3] Francisco Torrens,[4] and Juan A. Castillo-Garit[5,*]

Table of Contents

**Table S1**<br>Final machine learning model configurations implemented in WEKA 3.6	**2**
**Table S2**<br>Structural characteristics and main hyperparameters of the machine learning models used for QSAR classification.	**3**
**Table S3**<br>Molecular descriptors used in each machine-learning model developed in this study.	**4**
**Figure S1**<br>Representative consensus hits predicted as active by all four machine-learning models during the virtual screening. The figure shows 17 selected compounds (12 DrugBank molecules and 5 synthetic candidates) identified by the consensus modeling strategy	**6**

Table S1. Final machine learning model configurations implemented in WEKA 3.6.

Model	WEKA command-line configuration
**IBk**	weka.classifiers.lazy.IBk -K 3 -W 0 -A "weka.core.neighboursearch.LinearNNSearch -A \"weka.core.ManhattanDistance -R first-last\"" -t IBk_training.arff -T IBk_Test.arff -o
**J48**	weka.classifiers.trees.J48 -C 0.09 -M 5 -t J48_training.arff -T J48_Test.arff -o
**MLP**	weka.classifiers.functions.MultilayerPerceptron -L 1.0 -M 0.8 -N 500 -V 0 -S 0 -E 20 -H 13 -t MLP_training.arff -T MLP_Test.arff -o
**SMO**	weka.classifiers.functions.SMO -C 50.0 -L 0.001 -P 1.0E-12 -N 0 -V -1 -W 1 -K "weka.classifiers.functions.supportVector.RBFKernel -C 250007 -G 3.9" -t SVM_training.arff -T SVM_Test.arff -o

Table S2. Structural characteristics and main hyperparameters of the machine learning models used for QSAR classification.

Model	Algorithm structure	Key parameters	Distance / Kernel	Training settings
**IBk**	k-Nearest Neighbors classifier	k = 3 neighbors	Manhattan distance	LinearNNSearch algorithm
**J48**	Decision tree classifier	Confidence factor = 0.09; Minimum instances per leaf = 5	Not applicable	Pruned tree
**MLP**	Feed-forward artificial neural network	Hidden layer = 13 neurons	Not applicable	Learning rate = 1.0; Momentum = 0.8; Epochs = 500
**SMO**	Support Vector Machine	Cost parameter (C) = 50.0	RBF kernel (γ = 3.9)	Tolerance parameter = 0.001

Table S3. Molecular descriptors used in each machine-learning model developed in this study.

Dragon v 7.0.10			
**Model**	**Symbol**	**Definition**	**Class**
**MLP**	BLI	Kier benzene-likeliness index	topological descriptors
	SIC5	Structural Information Content index (neighborhood symmetry of 5-order)	Information indices
	X1Av	average valence connectivity index of order 1	Connectivity indices
	X3Av	average valence connectivity index of order 3	Connectivity indices
	SpMin4_Bh(m)	smallest eigenvalue n. 4 of Burden matrix / weighted by atomic masses	Burden eigenvalues
	SpMin1_Bh(e)	smallest eigenvalue n. 1 of Burden matrix / weighted by atomic Sanderson electronegativities	Burden eigenvalues
**IBk**	SpMax1_Bh(m)	highest eigenvalue n. 1 of Burden matrix / weighted by atomic masses	Burden eigenvalues
	SpMax1_Bh(p)	largest eigenvalue n. 1 of Burden matrix / weighted by atomic polarizabilities	Burden eigenvalues
	SpMin1_Bh(p**)**	smallest eigenvalue n. 1 of Burden matrix / weighted by atomic polarizabilities	Burden eigenvalues
	C-034	R--CR..X	Atom-centred fragments
	F05[O-F]	frequency of O - F at topological distance 05	2D frequency fingerprints
	F09[C-Cl]	Frequency of C - Cl at topological distance 9	2D frequency fingerprints
**J48**	Se	sum of atomic Sanderson electronegativities (scaled on Carbon atom)	Constitutional indices
	BLI	Kier benzene-likeliness index	Topological indices
	X0Av	average valence connectivity index of order	Connectivity indices
	Eig01_EA(dm)	Eigenvalue 01 from edge adj. matrix weighted by dipole moments	edge adjacency indices
	SpMin1_Bh(v)	smallest eigenvalue n. 1 of Burden matrix / weighted by atomic van der Waals volumes	Burden eigenvalues

	SpMin4_Bh(e**)**	smallest eigenvalue n. 4 of Burden matrix / weighted by atomic Sanderson electronegativities	Burden eigenvalues
	nCs	number of total secondary C(sp3)	Functional group counts
**SMO**	X2v	valence connectivity index of order 2	Connectivity indices
	CIC5	Complementary Information Content index (neighborhood symmetry of 5-order)	Information indices
	nN=C-N<	number of amidine derivatives	Functional group counts
	B09[O-P]	Presence/absence of O - P at topological distance 9	2D Atom Pairs
	nRNR2	number of tertiary amines (aliphatic)	Functional group counts
	F01[C-X]	Frequency of C - X at topological distance 1	2D Atom Pairs
	F05[O-F]	Frequency of O - F at topological distance 5	2D Atom Pairs

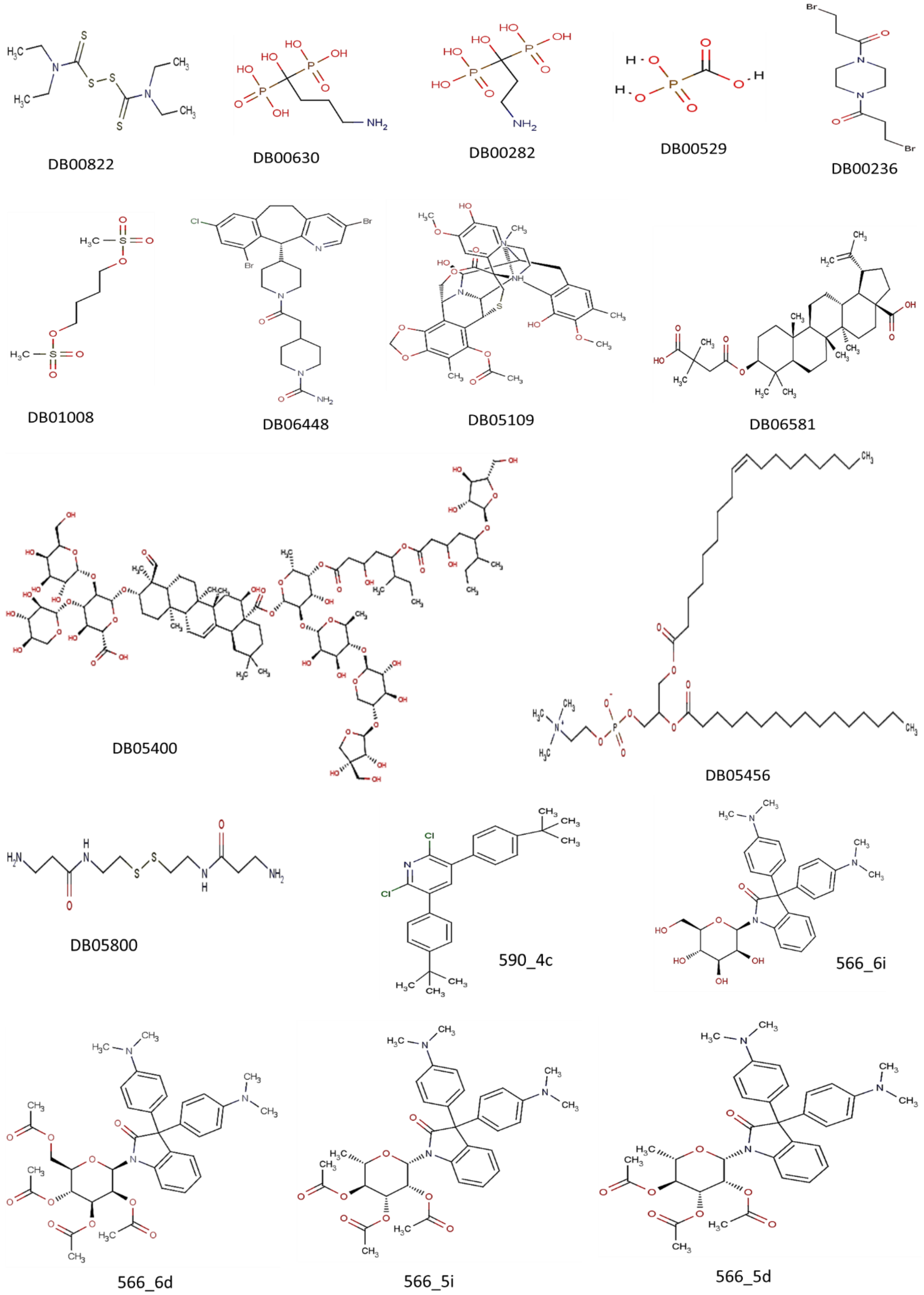

Figure S1. Representative consensus hits predicted as active by all four machine-learning models during the virtual screening. The figure shows 17 selected compounds (12 DrugBank molecules and 5 synthetic candidates) identified by the consensus modeling strategy.
